# Expression and Characterization of Recombinant, Tetrameric and Enzymatically Active Influenza Neuraminidase for the Setup of an Enzyme-Linked Lectin-Based Assay

**DOI:** 10.1371/journal.pone.0135474

**Published:** 2015-08-17

**Authors:** Marua Prevato, Ilaria Ferlenghi, Alessandra Bonci, Yasushi Uematsu, Giulia Anselmi, Fabiola Giusti, Sylvie Bertholet, Francois Legay, John Laird Telford, Ethan C. Settembre, Domenico Maione, Roberta Cozzi

**Affiliations:** 1 Research Center, Novartis Vaccines and Diagnostics s.r.l., (a GSK Company), Siena, Italy; 2 Department of Life Sciences, University of Siena, Siena, Italy; 3 Vaccine Research, Novartis Vaccines and Diagnostics, (a GSK Company), Basel, Switzerland; 4 Vaccine Research, Novartis Vaccines and Diagnostics Inc., (a GSK Company), Cambridge, MA, United States of America; US Food and Drug Administration, UNITED STATES

## Abstract

Developing a universal influenza vaccine that induces broad spectrum and longer-term immunity has become an important potentially achievable target in influenza vaccine research and development. Hemagglutinin (HA) and neuraminidase (NA) are the two major influenza virus antigens. Although antibody responses against influenza virus are mainly directed toward HA, NA is reported to be more genetically stable; hence NA-based vaccines have the potential to be effective for longer time periods. NA-specific immunity has been shown to limit the spread of influenza virus, thus reducing disease symptoms and providing cross-protection against heterosubtypic viruses in mouse challenge experiments.

The production of large quantities of highly pure and stable NA could be beneficial for the development of new antivirals, subunit-based vaccines, and novel diagnostic tools. In this study, recombinant NA (rNA) was produced in mammalian cells at high levels from both swine A/California/07/2009 (H1N1) and avian A/turkey/Turkey/01/2005 (H5N1) influenza viruses. Biochemical, structural, and immunological characterizations revealed that the soluble rNAs produced are tetrameric, enzymatically active and immunogenic, and finally they represent good alternatives to conventionally used sources of NA in the Enzyme-Linked Lectin Assay (ELLA).

## Introduction

Influenza virus infections affect mainly the upper respiratory tract and occasionally lung, and are responsible for high fever, cough, headache, muscle and joint pain. For young, elderly and chronically ill people, the disease could lead to more severe complications and sometimes to death [1]. According to WHO influenza fact sheet (2014), influenza epidemics cause annually 3 to 5 million cases of severe illness, and about 250 000 to 500 000 deaths worldwide.

Vaccination is the best way to prevent influenza virus infection and the potential complications of the associated diseases [1, 2]. Most currently licensed vaccines include the two surface glycoproteins that are the major influenza antigens, hemagglutinin (HA) and neuraminidase (NA). HA is the receptor-binding protein that mediates the attachment of the virus to the host cell receptors and mediates virus-cell fusion upon internalization [3, 4]. By contrast, NA with its sialidase activity acts as a receptor-destroying enzyme permitting transport of the virions through the mucus [5, 6] and allowing detachment of nascent virions from the host cell [3, 4, 7, 8].

The immune response against HA has been widely studied mainly because anti-HA humoral responses often includes neutralizing antibodies, which protect the host from viral infection [2, 9] Conversely, anti-NA antibodies are unable to protect the host from influenza virus infection, and immunity to NA has been accounted as “infection permissive immunity” [10]. Nevertheless, antibodies to NA can hamper virus penetration through the mucinous layer, favor recognition and clearance of infected cells by immune effector cells, as well as contribute to activation of complement–dependent cytotoxicity pathways [8, 9].

Therefore, induction of NA immunity would reduce influenza disease by limiting virus spread within the host, thus reducing morbidity and mortality, and decreasing viral shedding [8, 11]. Anti-NA immunity may be of particular importance when the HA of the circulating strain is a mismatch from those included in the vaccine and moreover when a new pandemic strain emerges containing a novel HA variant for which people are naive but where the NA is similar to those in circulating strains [7]. Despite all these potential benefits, licensed inactivated influenza vaccines are standardized based on a fixed HA amount [2, 8] while only recently vaccine designs have focused on NA.

NA inhibition (NI) titers are not routinely analyzed in vaccine trials [12] due in part to the fact that few serological assays are available to measure and characterize anti-NA responses. The two most used functional assays for the specific detection of NA inhibiting antibodies are the thiobarbituric acid assay (TBA) [13] and the Enzyme-Linked Lectin Assay (ELLA) [14, 15]. Both assays use fetuin as substrate of NA, but while the TBA assay is based on the chemical conversion of the free sialic acid to a chromogen, ELLA measures the residual terminal galactose exposed after fetuin desialylation using peanut agglutinin (PNA) for detection. The TBA assay requires handling of multiple hazardous chemicals and, thus ELLA is preferred; nevertheless the choice and the production of NA sources remain a challenge due to cost, time and availability issues.

Eukaryotic expression systems like insect cells [16–18] and yeast [19, 20] have already been exploited for the recombinant NA expression, although mammalian cells still remain the preferred expression systems because of their capacity to fold properly, assemble and post-translationally modify complex proteins [21].

NA is a tetramer of identical subunits, composed of several domains: the cytoplasmic domain, the transmembrane domain, the “head” active domain and the “stalk” domain that connects the head and the transmembrane domain. The head domain is highly conserved, while the stalk is the most variable region [22]. The available crystal structures of influenza neuraminidases from NA, of both A and B clades of influenza viruses, revealed that the NA ectodomain is a homotetramer, each monomer contains six antiparallel β-sheets forming a propeller-like arrangement [22].

Bosch and colleagues [23] demonstrated that the NA globular head can be expressed in HEK293 mammalian cells using a construct that included an N-terminal secretion sequence peptide, a purification tag and a tetramerization domain. Nevertheless, low yield, need for the generation of a stable cell line or for the improvement of the transient gene expression [24], and above all, costs are important issues that still need to be overcome to make recombinant NA (rNA) a tool for routine research.

Here, we describe the easy, rapid and high yield production of soluble, fully functional tetrameric, recombinant NA from both A/California/07/2009 (H1N1) and A/turkey/Turkey/1/2005 (H5N1) influenza virus strains. The method used combines the design, production and optimization of the NA-expressing vector in a new mammalian expression system designed to allow high-efficiency transfection of 293 human embryonic kidney cells grown in suspension culture at high cell density.

Functional and biochemical characterization of rNAs, in addition to 3D reconstruction of NA’s structure corroborated the successful production of correctly folded NA tetramers in the mammalian expression system, as alternative to NA purification from live virus or by baculovirus expression. Finally, these rNAs have been exploited as the NA source in an rNA-based functional ELLA assay.

## Material and Methods

### Ethics Statement

All animal studies were carried out in compliance with current Italian legislation on the care and use of animals in experimentation (Legislative Decree 116/92) and with the Novartis Animal Welfare Policy and Standards. Protocols were approved by the Italian Ministry of Health (authorization 249/2011-B) and by the local Novartis Animal Welfare Body (authorization AWB 201106).

### Materials

2′-(4-methylumbelliferyl)-α-d-N-acetylneuraminic acid (MuNANA), fetuin, carbonate/bicarbonate buffer, Tween 20, Bovine Serum Albumin (BSA), *Clostridium perfrigens* NA, horse radish peroxidase-labelled peanut agglutinin (HRP-PNA), 3,3′,5,5′-Tetramethylbenzidine (TMB), HCl, CelLytic M Reagent and Skim Milk were purchased from Sigma-Aldrich (St. Louis, MO). Polyclonal sheep sera specific for anti-N1 A/California/04/2009 (cat. N° 10/218), anti-N1 A/turkey/Turkey/1/2005 (cat. N° 8/126), anti-N1 A/NewCaledonia/20/99 (cat. N° 4/230), anti-N2 A/Wyoming/3/2003 (cat. N° 4/258), anti-NA B/Malaysia/2506/2004 (cat. N° 5/252), anti-NA B/Florida/4/2006 (cat. N° 9/316) were purchased from National Institute for Biological Standards and Control (NIBSC) (London, England). Influenza virus H1N1 A/California/04/2009 NA X-181 master seed was kindly provided by Novartis Flu Seed Facility (Basel, Switzerland).

### Construction of NA-expressing vectors

**Fig 1:**
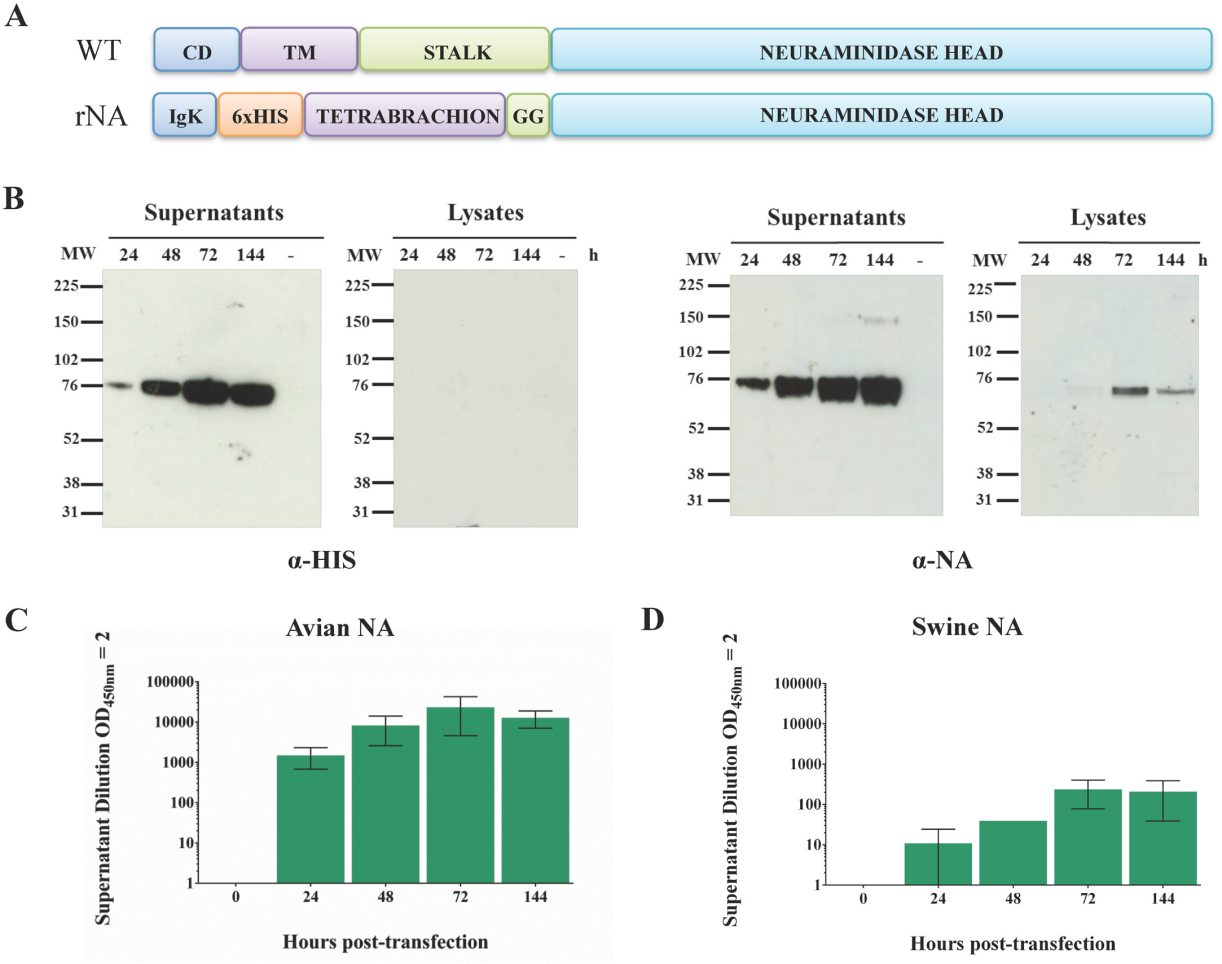
Design and expression of soluble, enzymatically active NA of H1N1 and H5N1 influenza viruses. (A) Schematic representation of wild type (WT) and recombinant NA (rNA). The cytoplasmic (CD), tetramerization (TM) and stalk domains of the WT NA were replaced by an Ig-k secretion sequence, a 6xHis-tag, and a tetrabrachion domain for protein tetramerization. (B) Reducing SDS-PAGE followed by WB of Expi293 supernatants and lysates collected every 24 h post-transfection. Anti-His tag (left) and anti-NA (right) stainings were used to specifically detect avian H5N1 rNA. (C, D) Titration of specific sialidase activity in culture supernatants harvested every 24 h post-transfection by ELLA and expressed as the supernatant dilution corresponding to an OD_450nm_ = 2. Data show mean±SD and are representative of at least three independent experiments.

Swine A/California/07/2009 (H1N1) (GenBank accession N° GQ377078.1) and avian A/turkey/Turkey/1/2005 (H5N1) (GenBank accession N° EF619973.1) NA are composed, from the N-terminal to the C-terminal, of a cytoplasmic domain (CD), a transmembrane and tetramerization domain (TM), a stalk region and a globular head domain that is responsible for their sialidase activity (Fig 1A, top). The nucleotide sequences of the globular heads plus the artificial N-terminal domain of 98 residues were human codon optimized for expression in mammalian cells and sub-cloned into prs5a vector (Novartis) using *Hind*III and *Not*I restriction enzymes. The artificial N-terminal domain contains a murine Ig κ-light chain secretion sequence (Igκ) followed by a 6 histidine purification tag (6xHis), a Tetrabranchion tetramerization domain (tetrabrachion) from the bacterium *Staphylothermus marinus* and two glycine residues as linker with the globular head sequence (Fig 1A, bottom). The identity of the resulting vectors was checked by sequencing.

### Expression and purification of rNAs

To produce rNAs, the expression vectors were transfected into Expi293F cells according to the manufacturer’s instructions (Life Technologies). Briefly, 30 μg of prs5a NA-expressing vectors were transfected into 30 ml culture containing 75 x 10^6^ Expi293F cells using ExpiFectamine 293 Reagent. Cells were incubated at 37°C, 120 rpm, 8% CO_2_ and after 24 h, ExpiFectamine 293 Transfection enhancer 1 and 2 were added. Cells were further incubated at 37°C for 144 h. Aliquots of cultures were harvested every 24 h and analyzed for NA expression and activity by Western Blot (WB) and ELLA, respectively. Seventy-two and 144 h after transfection, cell cultures were centrifuged at 1000 rpm for 7 min and the NA-containing supernatants were harvested, pooled, clarified by centrifugation, filtered through a 0.22 μm filter, and stored a 4°C until purification. Affinity chromatography with Ni^2+^ was used to purify rNAs from culture supernatants using HisTrap FF crude column (GE Healthcare). Fractions of interest were pooled and were concentrated by using 10 kDa cutoff spin concentrator (Millipore Amicon Ultra); sodium dodecyl sulphate-poly-acrylamide gel electrophoresis (SDS-PAGE) was performed to check purity. Tetrameric rNAs were purified by size exclusion chromatography (SEC) with a HiLoad Superdex columns (GE Healthcare) in a mobile phase containing Tris 50 mM, 150 mM NaCl pH8, CaCl_2_ 10 mM using Äkta Purifier (GE Healthcare). A high molecular weight standard kit (Biorad, 151–1901) containing Thyroglobulin (bovine) 670 kDa, γ-globulin (bovine) 158 kDa, Ovalbumin (chicken) 44 kDa, Myoglobin (horse) 17 kDa, and Vitamin B12 1.350 kDa was run to calibrate the columns and to estimate rNAs sizes. All the collected fractions were checked for NA content by SDS-PAGE and NA-containing fractions were pooled, aliquotated and stored at -20°C for further analysis.

### SDS-PAGE and western blotting

SDS-PAGE and WB analyses were performed to monitor NA expression in Expi293F cells transfected with p5RSa vectors, and to evaluate NA purity and identity during purification. Supernatants and pellets were obtained from centrifugation at 1200 rpm for 7 min of collected culture aliquots. Cells were lysed with CelLytic M Reagent at room temperature for 15 min and lysates were collected after centrifugation for 10 min at 8000 rpm, 4°C. Samples were mixed with 1X NuPage LDS loading buffer and 1X NuPage Sample reducing agent (Life Technologies), were heated at 100°C for 5 min and 20 μl were loaded onto a 4–12% NuPAGE Bis-Tris gel (Life Technologies). Novex Sharp Pre-stained Protein Standard or SeeBlue Prestained Standard markers were run onto each gel. In order to check the purity level of rNAs gels were stained with Comassie Blue while for rNA identity proteins were electro-transferred onto nitrocellulose membranes with iBlot 2 Dry Blotting System (Life Technologies). The membranes were blocked for 90 min with PBS + 0.1% Tween 20 + 5% milk and then incubated for 1 h with specific sheep polyclonal anti-N1 antibodies diluted 1:3000 or mouse anti-His-tag antibody diluted 1:1000 in blocking buffer.

After three washes in PBS + 0.1% Tween 20 (T-PBS) the membrane were incubated with HRP-labelled donkey anti-sheep or anti-mouse IgG at 1:5000 dilution, for 1 h at room temperature. Membranes were washed thrice with T-PBS, twice with PBS and then incubated with SuperSignal West Pico Chemiluminescent Substrate (ECL; Thermo Fisher Scientific, Rockford, IL) for 2 min. In the dark, photographic hyperfilms (GE Healthcare, Rydalmere, NSW, Australia) were placed against the membrane for increasing times and then developed using Curix60 (AGFA HealthCare, Greenville, USA) films processor.

### Peptide-N-glycosidase F (PNGase F) and endoglycosidase H (Endo H) treatment

For glycosylation analysis, N-linked oligosaccharides removal was carried out using recombinant PNGase F and Endo H (New England BioLabs, Beverly, Massachusetts) according to the manufacturer’s instruction. Briefly, 2.25 μg of purified swine A/California/07/2009 (H1N1) and avian A/turkey/Turkey/1/2005 (H5N1) rNAs were combined with 1 μl of 10X Glycoprotein Denaturing Buffer and then denaturated by heating reaction at 95°C for 5 min. Proteins were chilled on ice and centrifuged for 10 sec before adding 2 μl of 10X G7 Reaction Buffer, 2 μl 10% NP40, 6 μl H_2_O and 1 μl PNGase F to achieve a final volume of reaction of 20 μl. Samples were gently mixed and incubated at 37°C for 1 h. To assess the extent of deglycosylation, the proteins were mixed with 1X NuPage LDS loading buffer and 1X NuPage Sample reducing agent (Life Technologies), heated at 100°C for 5 min and finally loaded onto a 4–12% NuPAGE Bis-Tris gel (Life Technologies) subjected to SDS-PAGE. The mobility shifts were checked by Comassie staining.

### Transmission Electron Microscopy (TEM) on ghost membranes

5 μl aliquot of the purified rNA (0.6 mg/ml) sample was loaded onto glow-discharged (15 mA, 286 V) 300-square mesh copper grids coated with a thin carbon film (TED PELLA, Redding, CA) and let stand for 30 sec. Excess of solution was blotted by Whatman filter paper (Cat. N° 1001150). The grids were negatively stained with a solution of 1% buffered phosphotungsten acid (PTA) pH 7.2 for 30 sec. The excess of liquid was soaked off by Whatman filter paper. The grids were observed using a TEM Tecnai G2 spirit operating at voltage of 100 kV and a magnification of 87000X. Images were collected with a CCD camera Olympus SIS Morada 2K*4K.

### Image Processing and 3D Reconstruction

Images were first analyzed for defocus (1.1–3.2 μm) and Contrast Transfer Function (CTF) using the Medical Research Council (MRC) program CTFFIND3 [25]. Direct measurement of rNA tetramer diameters using command measure from the software EMAN [26, 27] clearly showed that rNA tetramers displayed similar external diameter corresponding to 100 Ǻ. Image phases were corrected for the effect of the contrast transfer function in IMAGIC5 [28] at a box of 64x64 pixels (corresponding to a 246x246 Ǻ box, 3.84 Ǻ/pixel step size in IMAGIC5). The different views of the tetramer were collected using the command “boxer” from the software EMAN. Images were then band pass filtered with a Gaussian edge at 17–200 Å to remove background noise and normalized. Filtered images were first pre-aligned interactively and subsequently using alignments with only limited angular ranges (-5°, +5°), finally vertical and translational alignments were performed. Centered rNA tetramers were than classified by Multivariate Statistical Analysis (MSA) to sort images into class average with similar features. The class averages obtained confirmed that rNA tetramers presented a small size variation and a typical C4 symmetry. The images were sorted into size groups by a combination of Multi Reference Alignment (MRA), MSA and classification applying the strategy described by White and coworkers [29]. After several iterations of MRA and MSA, the best class averages that contained the most of the raw data were used to generate 3D reconstruction. A total of 974 images where used to generate the 3D structure of the rNA tetramer. All the top-view class averages clearly showed rNA tetramers as structures made up by 4 subunits arranged around the same z-axis forming a ring like structure. The final 3-D was refined at 24 Å resolution (FSC = 0.5) [30] by iterating procedures of alignment and classification. 3D rendered surface was visualized in UCSF CHIMERA [31].

### Differential scanning fluorimetry (DSF)

To investigate the thermal stability of rNAs in presence of calcium ions, the NA protein solution was first mixed with 150 mM EDTA and then incubated at 25°C for 1 h. Then EDTA was removed from protein sample by buffer exchange (PD10 column, GE Healthcare), and different CaCl_2_ concentrations (from 0 to 20 mM) were added to the protein solution, followed by DSF analysis. Purified Avian H5N1 rNA was diluted to a final concentration of 20 μM in DSF buffer (25 mM Tris pH 8, 150 mM NaCl), containing different calcium concentrations. 4 μl of freshly prepared 100X solution of Sypro Orange from a 5000X stock (Invitrogen) were added to all protein solutions. The final volume of the reaction mixture was 40 μl in a 96-well plate (Thermo Fast 96-ABgene). The plate also included a baseline control containing Sypro Orange with DSF buffer only. The melting point (T_m_) of each protein-CaCl_2_ solutions was measured by ramping from 25°C to 95°C with 1°C per min increase. A quantitative PCR thermo cycler (Stratagene) has been applied to monitor and record the unfolding profile and the melting temperature (Ericsson, et al., 2006). The experiment has been performed as triplicated. Fluorescence intensities were plotted as a function of temperature and the reported T_m_ is the inflection point of the sigmoid curve.

### Kinetic parameters

The kinetic parameters of the purified rNAs were measured using a fluorescence-based assay that rely on the chemical conversion of the non-fluorescent MuNANA substrate into the fluorescent product 4-methylumbelliferone (4-MU) after NA action. Briefly, in a flat-bottom 96-well opaque black plate (Corning, Tewksbury, MA) serial dilutions, ranging from 0.59 μM to 600 μM, of MuNANA substrate were performed in 100 μl/well of reaction buffer (200 mM NaOAc, 20 mM CaCl_2_, pH 5.5, 0.01 mg/ml BSA) in duplicate. 100 μl/well of rNAs at 0.01 μg/ml (0.2 nM) concentration were added to all wells and plates were immediately incubated at 37°C. Fluorescence was measured every 10 min for 60 min with Infinite M200 Spectrophotometer (Tecan, Mannedorf, Switzerland) microplate reader using excitation and emission wavelengths of 355 nm and 450 nm, respectively. As a control, the highest MuNANA concentration was incubated without the enzyme. The background was calculated as the mean fluorescence of well incubated with reaction buffer only and was subtracted to all the other fluorescence values. Initial velocities were calculated as the difference of fluorescence values at T_3600”_ and T_0’_ versus time (3600”) and plotted against MuNANA concentration. The data were fitted to the Michaelis-Menten equation using non-linear regression analysis of data with GraphPad Prism 6.04. The best fits of the data produced the V_max_, K_m_, K_cat_, K_cat_/K_m_ values that are reported in Table 1.

**Table 1:** Enzymatic properties of swine H1N1 and avian H5N1 rNAs.

Enzyme	V_max_ (μM s^-1^)	K_m_ (μM)	K_m_ Std.	K_cat_ (s^-1^)	K_cat_ Std.	K_cat_/K_m_ (μM s^-1^)
**Swine H1N1**	6,116	29,82	1,913	30,58	0,5143	1,025
**Avian H5N1**	15,09	44,93	4,145	75,45	1,983	1,679

### Enzyme-Linked Lectin Assay

Recombinant NA ability to cleave N-acetylneuraminic acids from larger substrate was assayed according to ELLA protocol, previously described by Lambré et al. 1990. Briefly, Maxisorp Nunc 96well plates (Thermo Fisher Scientific) were coated with 7.5 μg/well of fetuin and incubated overnight at 4°C. Plates were washed 4X with 350 μl/well of T-PBS, covered and stored at 4°C at least for 1 month. Duplicates of rNA were serially diluted in incubation buffer (DPBS containing Ca^2+^ and Mg^2+^ and 1% BSA) and incubated on fetuin-coated plates overnight at 37°C. Serial dilutions of NA from *Clostridium perfrigens* were loaded on the same plate and used as internal positive control. Plates were washed 4X with 350 μl/well T-PBS and incubated with 100 μl of HRP-PNA. After 1 h incubation at room temperature, the plates were washed again and incubated with 100 μl/well of TMB for 30 min at room temperature. The reaction was stopped with 100 μl/well of 0.5 M HCl and absorbance was read at 450 nm using EnVision Multilabel Plate Reader (Perkin Elmer, Waltham, MA, USA). The standard dose of rNA that yields an OD_450nm_ = 2 was calculated and used for NI antibodies detection. Fifty-five μl/well of recombinant NA was added to 55 μl/well of heat-inactivated sera, serially diluted in incubation buffer in U-bottom 96-well plates. As virus positive control, 8 wells were incubated with rNA only. After 2 h incubation at 37°C, 100 μl/well were transferred into corresponding wells of a fetuin-coated plate and incubated overnight at 37°C. The plates were then washed and developed using the same procedure followed for NA activity titration. NI titers were defined as the reciprocal of serum dilution at which the mean absorbance was ≤ 50% of the mean signal of virus control (ID50); samples with a titer < 50 were assigned a value of 25. Data represents mean +/- SD of 3 independent experiments performed in duplicate.

### Production of rNA-specific antisera

All animal experiments were performed in accordance with Institutional Animal Care and Use Committee protocols and mice were housed in the Novartis Vaccines Animal Facility. 10 μg of either swine H1N1 or avian H5N1 rNA adjuvanted with MF59 in 100 μl of total volume were injected in female CD1 mice (5 weeks aged) three times intramuscularly. The second and the third shots were performed 30 and 45 days after the first dose, respectively. Pre-immune, post 2 (15 days after second dose) and post 3 (15 days after third dose) bleedings were collected for each mouse. To detect functional anti-NA antibodies the sera were pooled and inactivated for 30 min at 56°C.

### Statistical Analyses

All data were graphed using GraphPad Prism 6.04 software (GraphPad Software, La Jolla, CA, USA) and show mean and standard deviation. Parametric one-way ANOVA test was performed on selected groups considering a p-value of 0.05 or less statistically significant. ns: not significant, *** p<0.001, ** p<0.01, * p<0.05.

## Results

### High-level expression and purification of enzymatically active, soluble avian H5N1 and swine H1N1 recombinant NAs

**Fig 2:**
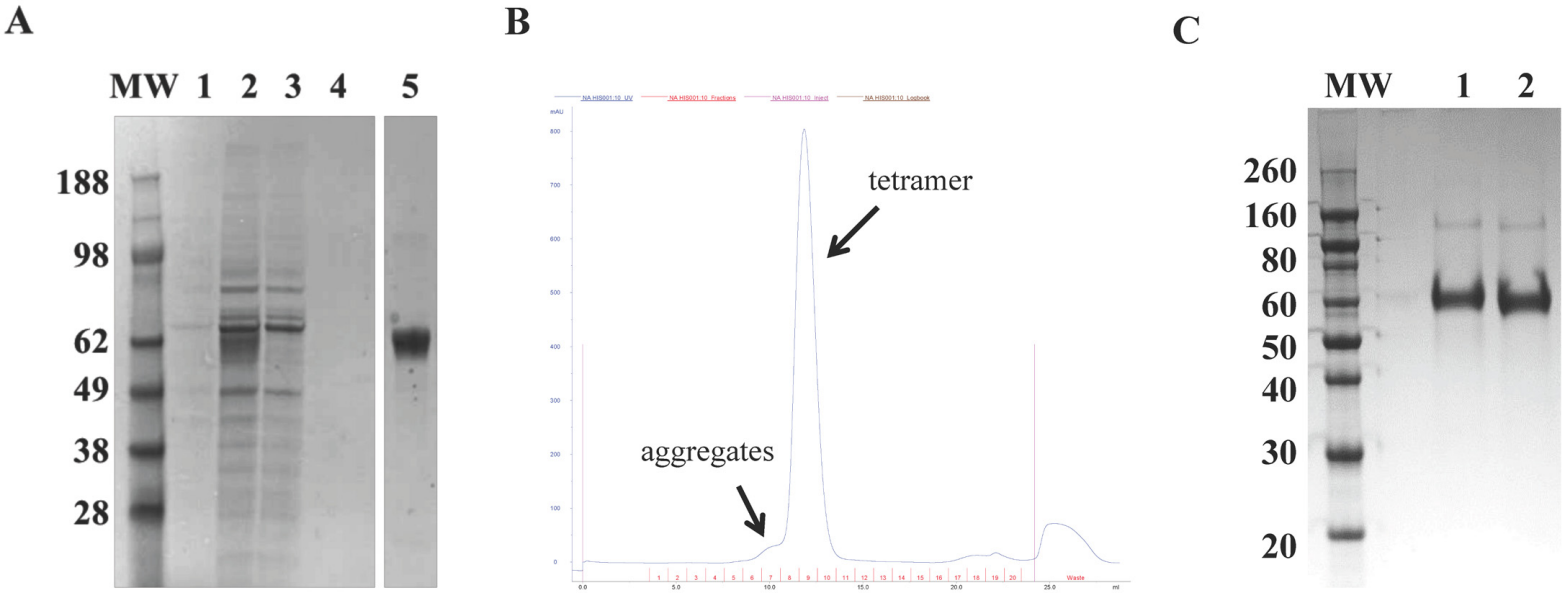
Purification of tetrameric swine H1N1 and avian H5N1 rNAs. (A) Avian H5N1 rNA purification by ion metal affinity chromatography; SDS-PAGE followed by Coomassie staining. MW: molecular weight marker (kDa); lane 1: pooled crude supernatants; lane 2: flow-through; lane 3: fraction eluted after washing with 10 mM imidazole; lane 4: fraction eluted after wash with 20 mM imidazole; lane 5: fraction eluted with 300 mM imidazole. (B) Gel filtration chromatogram of His-purified avian H5N1 rNA recorded at 280 nm wavelength. (C) SDS-PAGE followed by Coomassie staining of final purified, soluble, tetrameric swine H1N1 (lane 1) and avian H5N1 (lane 2) rNAs.Data shown are representative of at least three independent experiments.

The globular head domains of both swine A/California/07/2009 (H1N1) and avian A/turkey/Turkey/1/2005 (H5N1) NAs plus extra amino acids at the C-terminus of the stalk regions (amino acids 71–469 and 51–449, respectively) were fused to an artificial N-terminal stem composed of a secretion murine Ig-κ chain leader sequence, a 6xHis-tag, and a tetrabrachion tetramerization domain (Fig 1A). For high yield production, Expi293 mammalian cells were transfected with the pRS5a plasmid. The accumulation of secreted rNAs was characterized by measuring their expression and activity in cell lysates and culture supernatants at 24 h, 48 h, 72 h and 144 h post-transfection by WB (Fig 1B) and ELLA assay (Fig 1C and 1D), respectively. Both analyses revealed that rNAs expression increased over time and reached a plateau between 72 h and 144 h post-transfection. Soluble rNAs containing the 6xHis tag domain were purified from 72 h and 144 h pooled culture media using ion metal immobilized chromatography (Fig 2A), yielding 30 mg/L and 6 mg/L, of avian H5N1and swine H1N1 rNAs, respectively.

### rNAs form stable tetramers and are glycosylated

To confirm rNA assembly in a tetrameric structure, the purified rNAs were examined by gel filtration analysis and were shown to have a single sharp peak (Fig 2B). Based on the comparisons of the elution volumes (Ev) of both swine and avian rNAs with the Ev of molecular weight (MW) standards run in the same conditions, the calculated MW of these species consistent with a NA tetramer (≈240 kDa). No peaks indicative of rNA monomers, dimers, or trimers were detected during the SEC purification step. A minor amount of NA aggregates was represented by a small peak that eluted prior to the high molecular weight standard. The rNAs were obtained with a purity>90% by SDS-PAGE. (Fig 2C).

**Fig 3:**
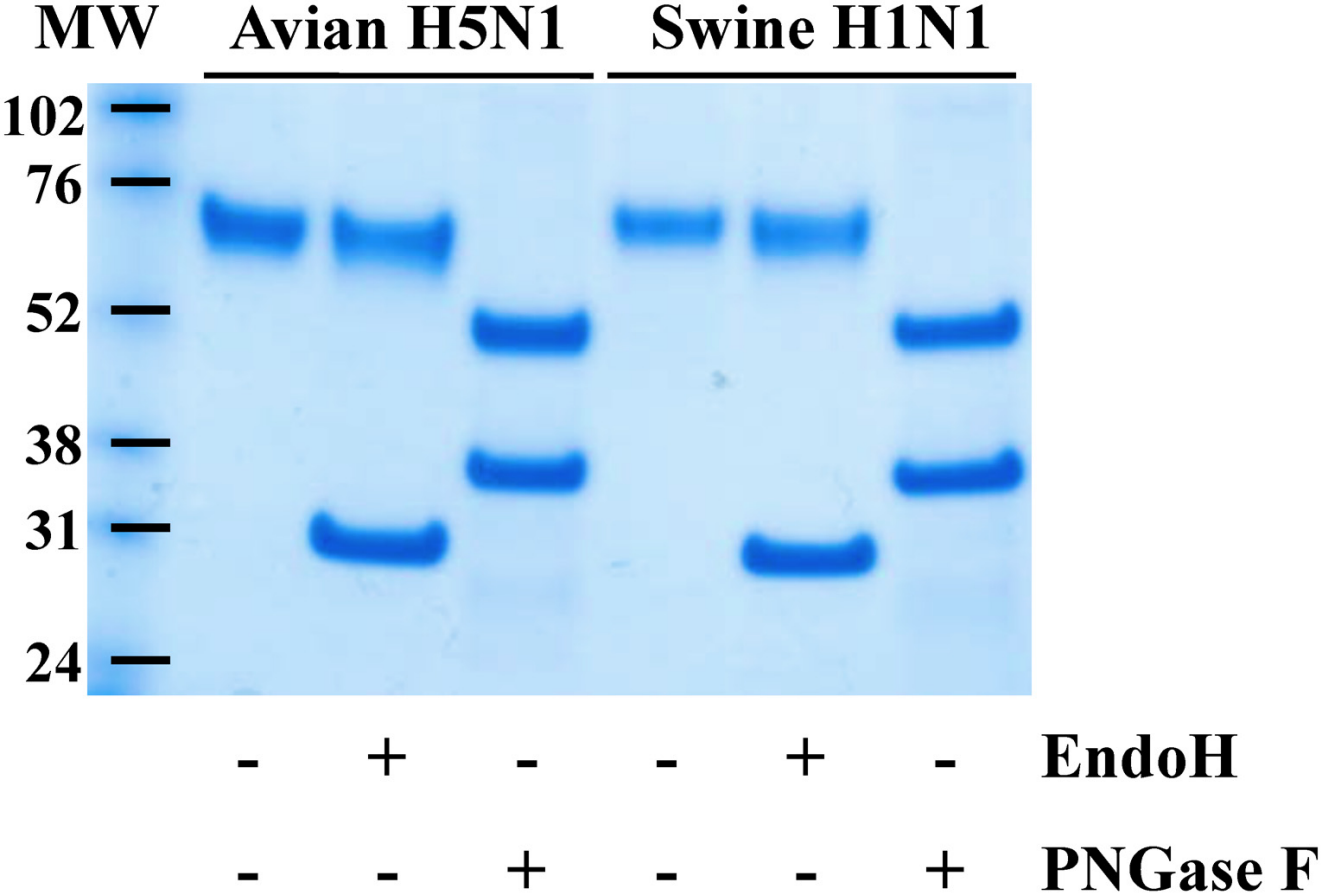
Glycosylation pattern of swine H1N1 and avian H5N1 rNAs. rNAs were deglycosylated with PNGase F or Endo H and molecular weights of treated and untreated samples were detected by SDS-PAGE followed by Coomassie staining. Data shown are representative of two independent experiments.

Mammalian cells are a suitable viral NA expression system, compared to baculovirus or yeast expression systems, because of the protein glycosylation patterns that are physiologically relevant in the context of human infections. Purified rNAs were deglycosylated with PNGase F or Endo H and their glycosylation patterns were analyzed by SDS-PAGE (Fig 3). Untreated NAs migrated at a MW of ≈70 kDa, higher than their theoretical MW of ≈52 kDa, suggesting post-translational modifications including glycosylation. PNGase F deglycosylated NAs ran at ≈50 kDa while Endo H treated NAs ran at ≈65 kDa. These data indicated that rNAs produced in mammalian cells contain N-linked glycosylations.

### Calcium binding induces a large increase in the stability of rNA

The several crystal structures of influenza neuraminidase show the presence of five calcium binding sites, one with high affinity close to the active site of each monomer and one with low affinity on the molecular 4-fold symmetry axis [32]. Calcium binding has been reported to be important for NA enzymatic activity and thermostability [33].

**Fig 4:**
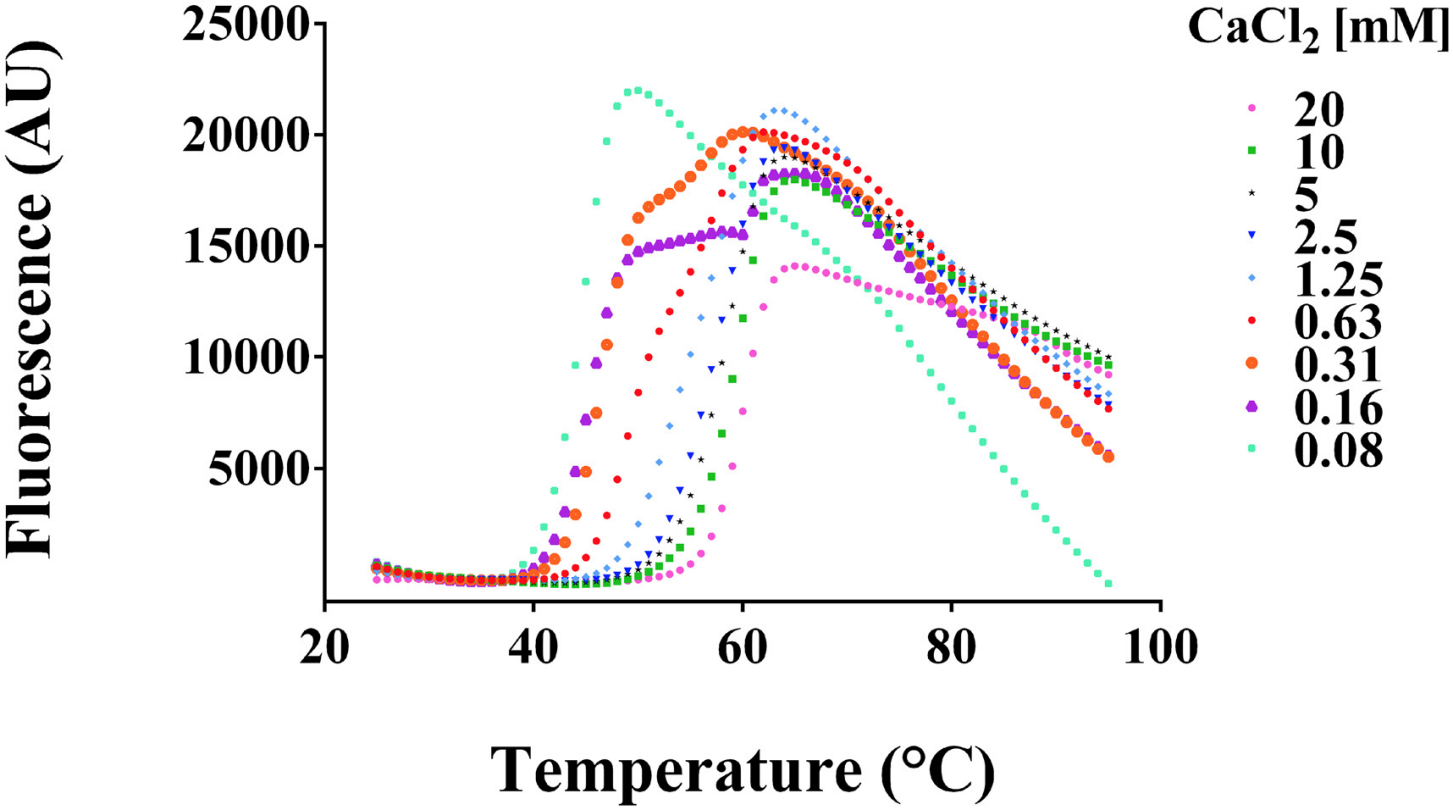
DSF analysis of avian H5N1 rNA. The thermostabilizing effect of Ca^2+^ ions binding to rNA was detected by DSF in the presence of Sypro orange. The graph shows fluorescence intensity *vs* temperature for increasing amount of Ca^2+^, 0.079–20 mM, in 25 mM Tris pH 8, 150 mM NaCl buffer.

The thermostability of A/turkey/Turkey/1/2005 (H5N1) rNA was measured by DSF using Sypro Orange as the external fluorescent probe. The thermostabilizing effect of Ca^2+^ binding to avian rNA was investigated by incubating the purified protein with increasing concentrations of Ca^2+^. A T_m_ shift from 44°C to 59°C was observed as the Ca^2+^ concentration in the solution was increased (Fig 4). This result indicated that higher concentrations of Ca^2+^ contribute to the NA thermal stability.

### Soluble, tetrameric rNAs are enzymatically active

**Fig 5:**
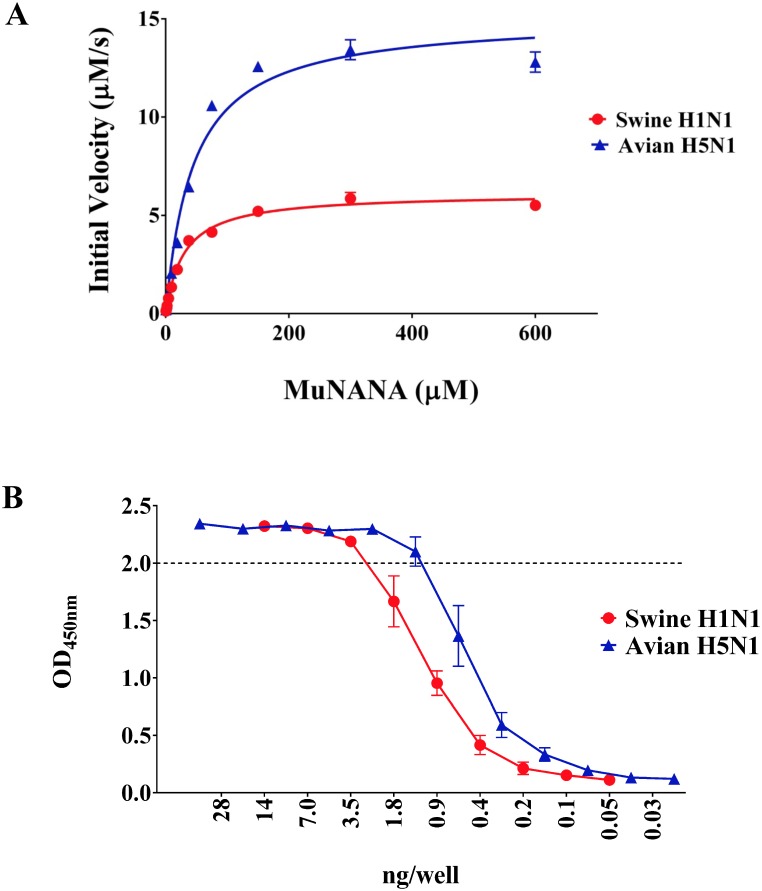
Sialidase activity of swine H1N1 and avian H5N1 rNAs. (A) Kinetic analyses of rNAs. Triplicate data sets for each experiment were used to calculate the steady-state velocity at decreasing concentrations of MuNANA substrate for each enzyme, and were expressed as initial rates (μM/s) *vs* concentration of substrate. The reactions containing 0.2 nM of enzyme and 0.59–600 μM of MuNANA were performed at 37°C in 200 mM NaOAc, 20 mM CaCl_2_, 0,01 mg/ml BSA, pH5.5. (B) Titration of rNAs activity by ELLA. Decreasing amount of rNAs were incubated with a fixed amount of fetuin overnight at 37°C and OD detected were graphed *vs* the protein concentration. Data represent mean±SD of 3 independent experiments performed in duplicate.

To determine and compare the specific activity of both swine H1N1 and avian H5N1 rNAs, a MuNANA activity assay was performed calculating the Michaelis-Menten steady state kinetic constants (K_m_, K_cat_, K_cat_/K_m_) (Fig 5A and Table 1). As previously reported [34], the kinetic parameters for the two rNAs were substantially different. The rNA derived from the avian H5N1 at 0.2 nM, corresponding to 0.01 μg/ml, catalyzed more efficiently the MuNANA substrate than swine H1N1 rNA at the same concentration, as indicated by the K_cat_/K_m_ ratios of 1.679 μM s^-1^ and 1.025 μM s^-1^, respectively. In addition, avian H5N1 rNA V_max_ was 15.09 μM s^-1^, higher than the swine H1N1 rNA that had a V_max_ of 6.116 μM s^-1^. Interestingly, the affinity of the avian rNA for the MuNANA substrate was lower than the swine rNA, as demonstrated by the K_m_ constants of 44.93 μM and 29.82 μM, respectively.

Next, the activity of both purified rNAs was compared using fetuin, a larger substrate containing N-acetylneuraminic acid, used in the ELLA assay. Avian H5N1 rNA was more active than swine H1N1 rNA (Fig 5B), judging from the amounts of rNAs that yielded an OD_450 nm_ = 2, in agreement with the data obtained by MuNANA assay.

### Visualization and structural features in 3D reconstructions of recombinant NAs

**Fig 6:**
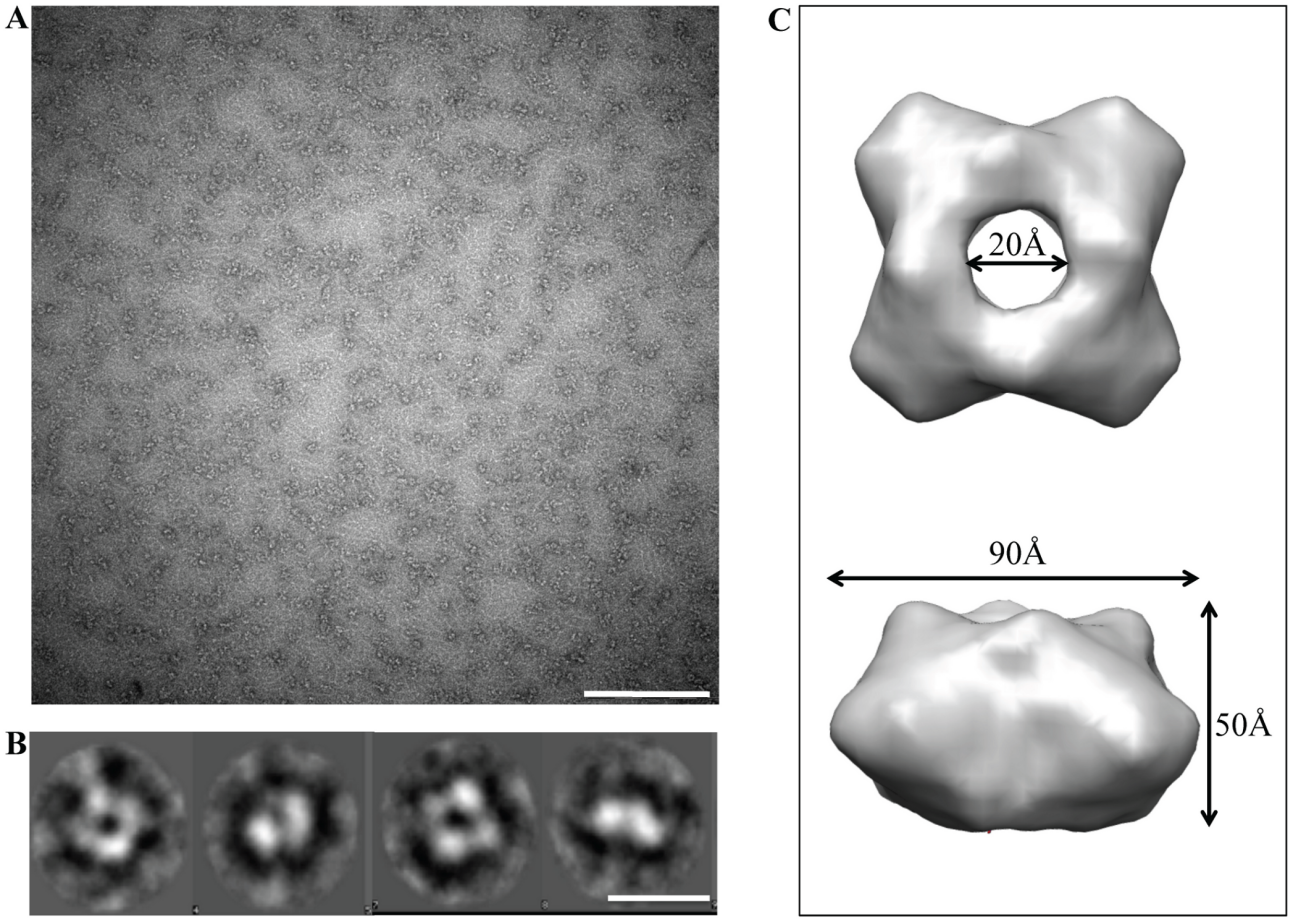
Structural characterization of avian H5N1 rNA using TEM with single particles reconstruction. (A) Typical negative staining TEM image of avian H5N1 rNA. Scale bar corresponds to 200 nm. (B) Representative class averages of avian H5N1 rNa tetramers. Each class average (~50 images per class) contains particles selected from several micrographs and represents the different orientations of the enzyme. Scale bar corresponds to 10 nm. (C) Top (upper panel) and side (lower panel) surface views of the reconstructed avian H5N1 rNA globular head obtained at a resolution of 24 Å (FSC = 0.5).

An additional confirmation that recombinant NA forms stable tetramers in solution was obtained by visualizing the purified protein using negative stain TEM. As shown in Fig 6A, avian H5N1 rNA sample appeared as differentially oriented homogeneous population of ring-like structures, with a uniform external diameter of 90 Å, an internal diameter of 20 Å and a height of 50 Å. Single particle reconstruction method was applied to TEM images in order to generate the three-dimensional structure of the tetrameric head. Single boxed rNAs tetramers (box size 64x64 pixel) [26, 27] (Fig 6B, top) were firstly band pass filtered in order to increase the signal-to noise ratio, than rotationally and translationally aligned, and finally centered before undergoing MSA for classification [28, 29]. Fig 6B shows a selection of rNA tetramers class averages, representative of the different orientations of the oligomer particle on the carbon film support. The 3D-EM structure (Fig 6C) [31] of the soluble tetrameric head generated confirm to be a ring-like structure composed by four lobes and presenting an internal hole of 30 Å in diameter. The reference-free reconstruction obtained without imposing tetrameric symmetry, shows a clear C4 symmetry, already visible in the class-averages.

### Both swine and avian rNAs are suitable sources of NA in ELLA

**Fig 7:**
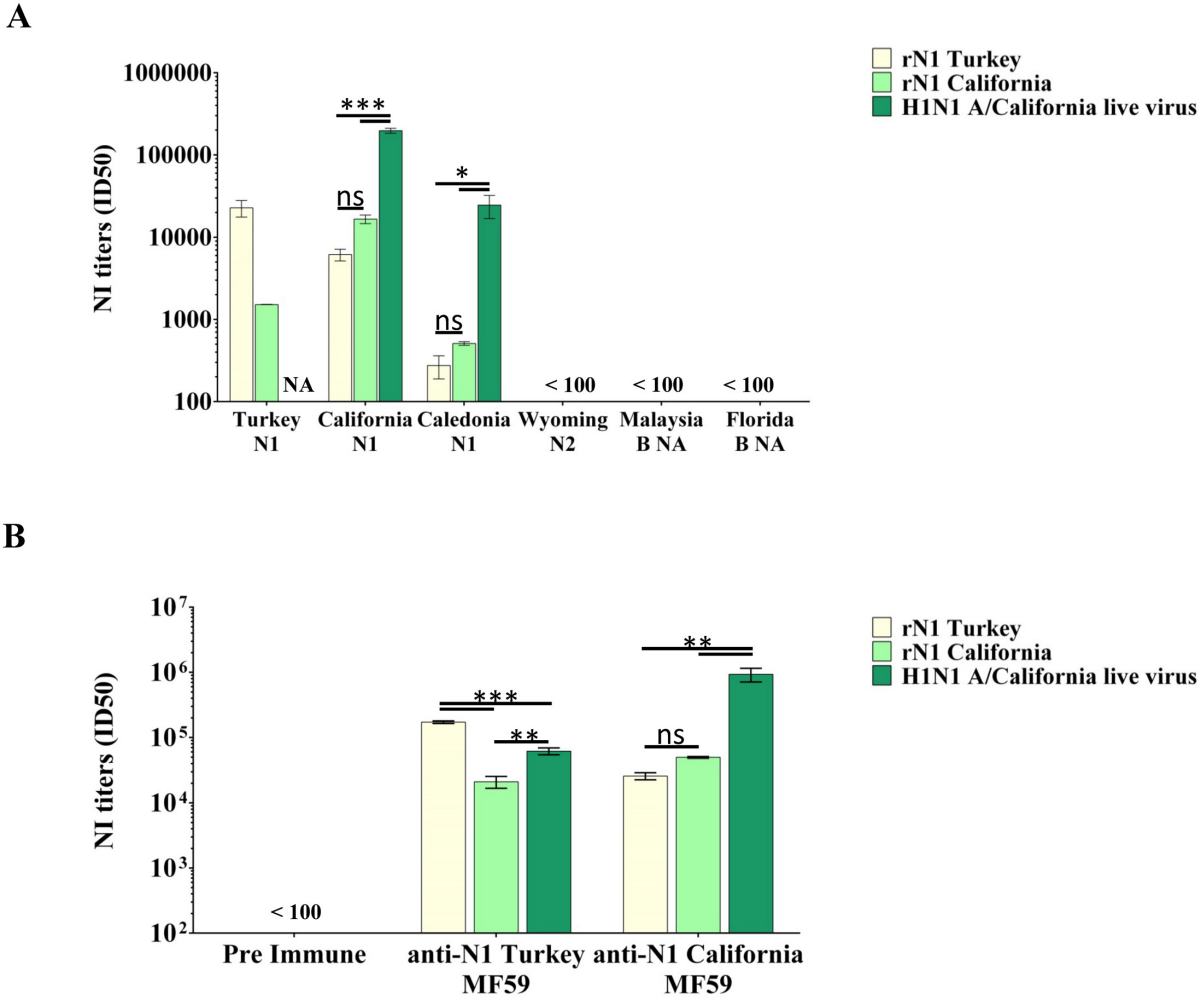
rNAs as sources of sialidase in ELLA. (A) NI titers determined in a panel of NIBSC sheep polyclonal sera specific for A/turkey/Turkey/01/2005, A/California/07/2009 and A/Caledonia/22/99 N1, A/Wyoming/3/2003 N2, and B/Malaysia/2506/2004 and B/Florida/4/2006 B NAs. (B) NI titers in sera of mice immunized with swine H1N1 and avian H5N1 rNAs adjuvanted with MF59. Data show mean±SD from three independent experiments performed in duplicate. NA = not assayed.

To investigate the suitability of soluble swine H1N1 and avian H5N1 rNAs as sources of enzyme in ELLA inhibition test, a standard dose of NA corresponding to an OD_450nm_ = 2 was calculated from the titration curve (Fig 5B) and incubated with serial dilutions of a panel of sheep polyclonal sera specific for different NAs. Swine H1N1 and avian H5N1 rNA’s activity were specifically inhibited by sera raised against the homologous N1 (Fig 7A). Furthermore, functional cross-reactive antibodies were detected in sera raised against heterologous A/N1, but not against A/N2 or B/NA. The strength of antibody cross-reactivity correlated with the degree of identity between the different A/N1 strains (S1 Fig). NI titers measured using the whole live A/California/07/2009 (H1N1) virus as source of NA followed the same trend.

### rNAs are immunogenic in mice

To investigate whether the rNAs were also able to elicit antibodies, sera obtained from administration of swine H1N1 and avian H5N1 rNAs adjuvanted with MF59 to mice were analyzed by ELLA using either rNAs or the whole A/California/07/2009 (H1N1) live virus as the NA source. Fig 7B shows that specific NA activity inhibition was measured after the second shot in mouse sera raised against the homologous NA, and functional cross-reactive antibody to both rNAs were also detected. The functional anti-NA responses reached a plateau after the second dose. The NI titers obtained with rNAs were comparable to those measured using whole live virus as the NA source, suggesting that the soluble, tetrameric, fully active rNAs possess the fundamental characteristics of the native NA normally displayed on the virus surface.

## Discussion

In this work, we describe a simple, rapid, and scalable procedure resulting in high yields of functionally-active tetrameric NA from both the 2009 pandemic H1N1 and 2005 avian H5N1 influenza strains. To obtain soluble, fully functional, tetrameric rNAs, the cytoplasmic tail, the transmembrane domain, and part of the stalk were replaced with an artificial N-terminal structure. A major challenge in producing recombinant rNA is the inability of the head domain to fold and tetramerize as an independent unit. To date, many tetramerization domains have been added to the NA head to ensure correct folding and tetramerization. A recent study described a generic procedure for the expression of NAs in a baculovirus expression system, comparing two different artificial tetramerization domains: the GCN4-pLI from yeast and the tetrabrachion domain from *Staphylothermus marinus* [35]. The tetrabrachion domain added at the NA N-terminus was superior to the GCN4-pLI containing construct, and demonstrated good solubility, increased stability, and biochemical properties closer to the native viral NA [35]. Based on these data, we selected the tetrabrachion domain as the artificial domain to stabilize the rNAs. In addition, to enable specific purification from culture supernatant, an Ig k-light chain secretion sequence and a His-tag purification domain were inserted before the tetrabrachion protein tetramerization motif (Fig 1A).

The transfection of mammalian of Expi293 human embryonic kidney cells, performed according to the manufacturer protocol, followed by a simple purification process of rNAs from culture supernatants yielded highly pure, correctly folded recombinant NAs tetramers, as determined by SEC analysis and EM 3D-reconstruction. Yields of 30 mg/L and 6 mg/L, for avian and swine rNA were achieved, respectively, and were 15 to 50 times higher than those obtained using other mammalian cells [24] or eukaryotic expression systems [19, 35].

To biochemically characterize the rNAs, stability and specific enzymatic activity were assayed using isothermal titration calorimetry and standard MuNANA assay. DSF experiments revealed that in presence of 10 mM Ca^2+^ avian rNA is stable up to 59°C. This result strongly supports the evidence that enzymatic activity is augmented in the presence of high Ca^2+^concentrations [32]. Moreover, our data support the finding by Lawrenz M. et al. (2010) who demonstrated a consistent structural role of Ca^2+^ in the stabilization of N1 active site by using molecular dynamics simulations. The kinetics of avian and swine rNAs enzymatic activity were comparable to those of native NA on the virus surface (Abed et. al, 2013). Moreover, the difference in activity between the two rNAs is in agreement with previous evidence showing a lower activity for A/California/07/2009 N1 compared to other swine and human N1 and N2 [34].

We then tested our rNAs in functional NA inhibition assays. Although NA-inhibiting antibodies that bind in close proximity to the catalytic site could be detected with MuNANA-based inhibition assays, the small size of MuNANA substrate allows the binding to the catalytic site even in the presence of antibodies that would block contact with larger substrates, like fetuin [36]. This could lead to an underestimation of the total functional NI antibodies [37]. Thus, in our study rNAs functional inhibition was directly tested in ELLA, that use fetuin as substrate. Specific NA activity was detected for both avian and swine rNAs with A/turkey/Turkey/01/2005 rN1 being more active than A/California/07/2009 rN1, in agreement with data generated with the MuNANA assay. Enzymatic activity of rNAs was specifically inhibited by a panel of commercial homologous and heterologous anti-A/N1 sera. NI titers were not observed with A/N2 and B NA antisera. All clade A influenza virus N1s tested share more than 80% identity, while A/N2 and B NAs share less than 35% identity with A/N1s (S1 Fig). This may explain why recombinant N1 NAs used as the NA source measures cross-reactive NI titers in anti-N1 A/California/07/2004, A/NewCaledonia/20/99 and A/turkey/Turkey/01/2005 specific sera but not in sera raised against distant NAs. It is noticeable that rNA-based ELLA NI titers are lower than those measured using the whole A/California/07/2009 (H1N1) live virus as source of NA. This could be due to the lack of part of the stalk and N-terminal regions in the rNAs. However, whole live viruses could be difficult to obtain, propagate and manage, and could require BLS-2 or higher level of containments, especially when highly pathogenic. The whole live virus also contains the HA antigen and suffers of interference issues due to steric hindrance of anti-HA antibodies that could be present in the sera. Reassortant viruses containing the NA of interest and a mismatched HA [38] as well as detergent treated viruses [39] have been widely used as surrogates of the whole live virus in ELLA but however still suffer of several limitations. These approaches are based on expensive, laborious and time-consuming procedures as well as infectious reagents. Conversely, recombinant NAs are safer, easier to produce, characterize and handle, and moreover large amount of rNAs could be produced in less than two weeks.

The activity of rNAs and their specific inhibition in ELLA proved rNA as suitable and convenient sources of NA.

Very high specific and cross-reactive NI titers were measured in sera of mice that were injected with both swine and avian rNAs adjuvanted with MF59, using either rNAs and the whole A/California/07/2009 (H1N1) live virus as the NA source. Although the NI titers measured using the whole live virus were higher than those measured using the rNAs, these data confirm previous evidences that recombinantly produced NAs are still able to stimulate the humoral immune response.

In summary, this work describes a rapid method for the production of soluble, tetrameric and glycosylated recombinant neuraminidase using optimized NA-expressing vectors in mammalian cells. The rNAs are enzymatically active and able to induce functional antibodies, demonstrating their potential as a tool for the screening of new NA inhibitors, for the development of novel diagnostic tools, and for structural studies, as a non-exhaustive list of possible applications. The development of standardized ELLA based on these enzymatically active rNA might allow routine NA immunity analysis in standard influenza vaccine clinical trials enabling better characterization of the immune response during vaccination or infection.

## Supporting Information

S1 Fig Multiple sequence alignment of influenza neuraminidases. Structure-based amino acid sequence alignment of the whole A/turkey/Turkey/01/2005, A/California/07/2009 and A/Caledonia/22/99 N1 NAs, A/Wyoming/3/2003 N2 NA and B/Malaysia/2506/2004 and B/Florida/4/2006 B NAs. Secondary structure elements refer to the crystal structure of the A/Vietnam/1203/04 (H5N1) NA globular head (PDB 2HTY). Identical residues are shown with a red background, whereas similar residues are shown in red and highlighted with blue boxes (http://espript.ibcp.fr/ESPript/ESPript/).
(TIF)
