# Supplementary figures and images for: Expression and Characterization of Recombinant, Tetrameric and Enzymatically Active Influenza Neuraminidase for the Setup of an Enzyme-Linked Lectin-Based Assay
Source: PLoS One. 2015 Aug 17;10(8):e0135474. doi: 10.1371/journal.pone.0135474 (PMC4539205; doi:10.1371/journal.pone.0135474)

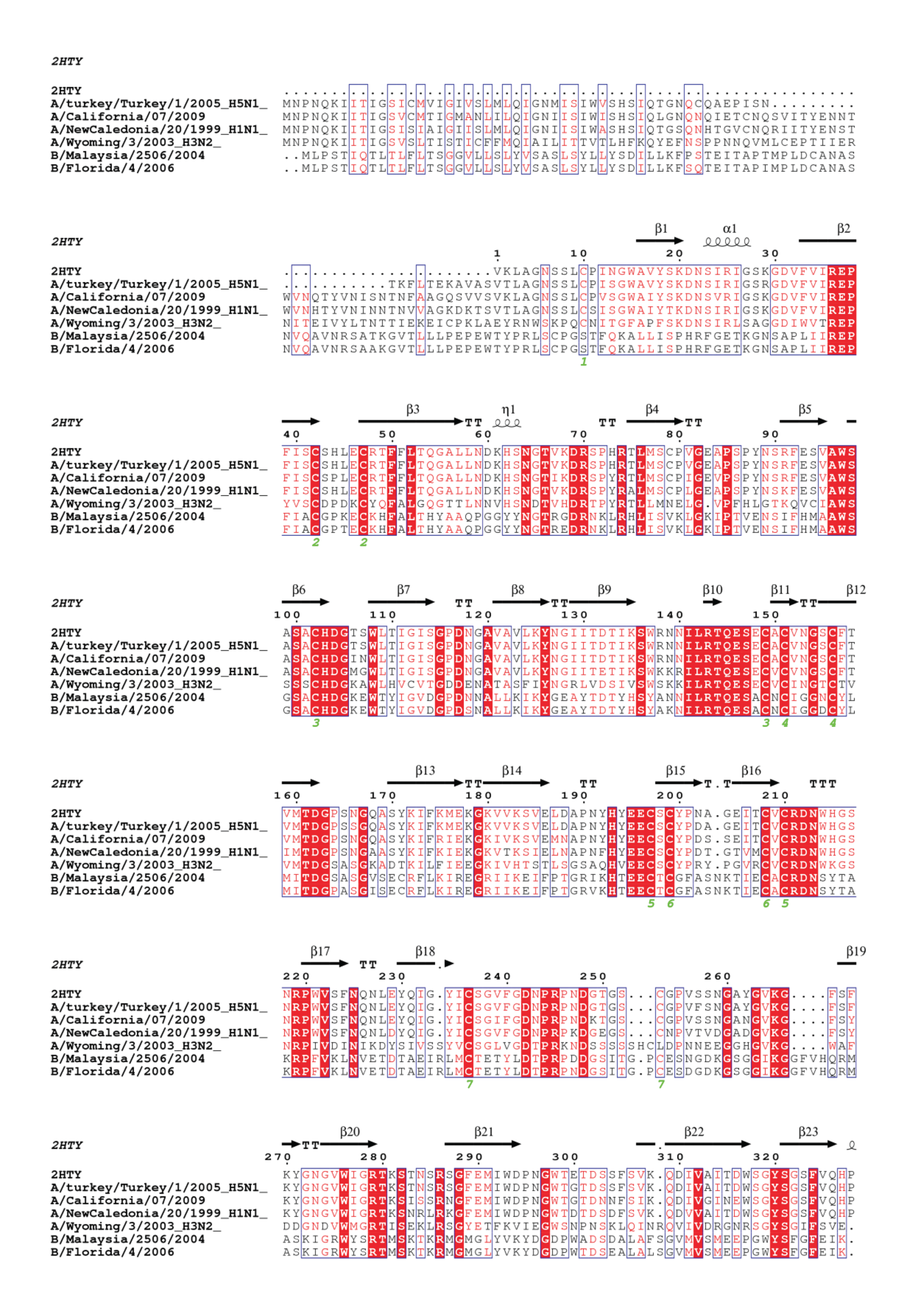

Supplement: S1 Fig — Structure-based amino acid sequence alignment of the whole A/turkey/Turkey/01/2005, A/California/07/2009 and A/Caledonia/22/99 N1 NAs, A/Wyoming/3/2003 N2 NA and B/Malaysia/2506/2004 and B/Florida/4/2006 B NAs. Secondary structure elements refer to the crystal structure of the A/Vietnam/1203/04 (H5N1) NA globular head (PDB 2HTY). Identical residues are shown with a red background, whereas similar residues are shown in red and highlighted with blue boxes (http://espript.ibcp.fr/ESPript/ESPript/). (TIF) [file pone.0135474.s001.tif]
